# Potential of antithrombin III as a biomarker of antidepressive effect in major depressive disorder

**DOI:** 10.1192/j.eurpsy.2021.310

**Published:** 2021-08-13

**Authors:** R. Song, Y. Shi

**Affiliations:** Department Of Neurology, Affiliated ZhongDa Hospital, School of Medicine, Southeast University, Nanjing, China

**Keywords:** major depressive disorder, antithrombin III, occipital repetitive transcranial magnetic stimulation antidepressive effect, biomarker

## Abstract

**Introduction:**

Previous study has identified increased antithrombin III (ATIII) in patients with major depressive disorder (MDD), supporting ATIII as a potential biomarker for depression diagnosis.

**Objectives:**

This study aimed to reveal the alteration of ATIII after occipital repetitive transcranial magnetic stimulation (rTMS), and illuminate its power to evaluate and predict the curative effects in MDD treatment.

**Methods:**

A total of 90 MDD patients were recruited and further intervened with rTMS in occipital for individualized, standard or sham treatment for five days. Those of 74 patients underwent entire detection, including clinical assessments, blood collection and protein measurement.

**Results:**

After treatment, decreased ATIII were detected in both the individualized and the standard group (p=0.000 and 0.001, respectively) instead of the sham one. Especially, the reduction in ATIII in the individualized group was associated with improvements in several neuropsychological assessments. Besides, ATIII at baseline in the standard group and after the individualized rTMS showed high performance to evaluate or predict the response to the 5-day treatment (AUC=0.771, 95%CI, 0.571-0.971; AUC=0.875, 95%CI, 0.714-1.000, respectively) and the remission in follow-up (AUC=0.736, 95%CI, 0.529-0.943; AUC=0.828, 95%CI, 0.656-1.000, respectively). Furthermore, both baseline ATIII and change in ATIII involved in the prediction of 24-item Hamilton Depression Rating Scale in the follow-up study with significant predictive values (p=0.0240 and 0.0233, respectively).

**Conclusions:**

This study detected a reduction in ATIII after occipital rTMS, further revealed the relationships between change in ATIII and therapeutic response, and ultimately provided evidence for the potential of ATIII as a biomarker for the evaluation and prediction of antidepressive effect.

**Disclosure:**

No significant relationships.

